# Association between cumulative blood pressure in early adulthood and right ventricular structure and function in middle age: The CARDIA study

**DOI:** 10.1002/clc.23763

**Published:** 2022-01-03

**Authors:** Shenrong Liu, Yanfen Liao, Zongyuan Zhu, Shushui Wang, Yifan Li, Dongpo Liang, Yumei Xie, Zhiwei Zhang

**Affiliations:** ^1^ Department of Cardiac Pediatrics, Guangdong Cardiovascular Institute Guangdong Provincial People's Hospital/Guangdong Academy of Medical Sciences Guangzhou China; ^2^ Department of Stomatology The First Affiliated Hospital of Sun Yat‐sen University Guangzhou China; ^3^ Department of Huiqiao Building, Nanfang Hospital Southern Medical University Guangzhou China

**Keywords:** blood pressure, right ventricular structure and function, young adult

## Abstract

**Objective:**

Cumulative blood pressure (BP) exposure is a known risk factor for cardiovascular disease. This study sought to investigate the association between cumulative BP from early adulthood to middle age and right ventricular (RV) structure and function in middle age.

**Methods:**

We included 2844 participants from the CARDIA study (Coronary Artery Risk Development in Young Adults). Cumulative BP over the 30‐years follow‐up was defined as the sum of the product of mean BP for each pair of consecutive examinations and the time interval between these two consecutive examinations in years. RV structure and function were assessed by echocardiography. The main analyses utilized logistic and linear regression models.

**Results:**

In fully adjusted models, higher cumulative systolic BP was independently associated with lower tricuspid annular plane systolic excursion (TAPSE), right ventricular peak systolic velocity (RVS′), right ventricular early diastolic velocity (RVe′), and higher pulmonary arterial systolic pressure. Higher cumulative diastolic BP was independently associated with smaller RV basal diameter, lower TAPSE, RVS′, and RVe′. For categorical analyses of RV dysfunction, cumulative systolic BP was not related to systolic dysfunction. Per 1‐SD increase in cumulative systolic BP was associated with a higher risk of diastolic dysfunction, while an increase in cumulative diastolic BP was associated with a higher risk of systolic dysfunction and diastolic dysfunction.

**Conclusions:**

Cumulative exposure to increased BP from early adulthood to middle age was associated with incipient RV systolic and diastolic dysfunction in middle age. Exposure to higher diastolic BP levels from early adulthood to middle age was associated with a smaller RV basal diameter in middle age.

## 1 INTRODUCTION

High blood pressure (BP) is a known risk factor for cardiovascular disease (CVD).^1^, ^2^ To date, CVD risk prediction algorithms have predominantly focused on BP at a single time measurement, and do not consider the potential effect of long‐term BP exposure.^3^, ^4^ Cumulative BP exposure, a measure which incorporates both BP level and exposure time, has provided incremental prognostic value and improvement in CVD risk classification.^5^, ^6^

In recent years, with the advent of reliable and reproducible echocardiographic measures of RV function, right ventricular dysfunction is being increasingly recognized in hypertension^7^, ^8^ and found to be an independent predictor of adverse cardiovascular outcome.^9^, ^10^ Previous studies suggested that subclinical RV dysfunction may be observed early in the course of arterial hypertension.^11^, ^12^, ^13^, ^14^ However, these studies have been limited by either their small sample size or cross‐sectional nature. The association of cumulative BP exposure from early adulthood onwards with RV structure and function in later life has not been studied.

Using longitudinal data from the Coronary Artery Risk Development in Young Adults (CARDIA) study, we aimed to evaluate the association of cumulative BP exposure starting during early adulthood (ages 18–30 years) with several echocardiographic indices of RV myocardial structure and function in later life.

## 2 METHOD

### 2.1 Study population

The CARDIA study is a longitudinal cohort that was designed to study determinants of subclinical and overt CVD in 5115 black and white young adults initially aged 18–30 years in 1985–1986 across four cities (Birmingham, AL; Chicago, IL; Minneapolis, MN; and Oakland, CA). Detailed descriptions of the design, recruitment, and protocol for examinations have been described previously.^15^ Follow‐up visits were conducted at years 2, 5, 7, 10, 15, 25, and 30 after baseline and retention rates among surviving participants at each visit were 90.5%, 85.7% 80.6%, 78.5%, 73.6%, 71.9%, 72.1%, and 71.0%, respectively. The CARDIA study was approved by the Institutional Review Boards at each study site, and written informed consent was obtained from participants at all examinations. The authors obtained permission from the CARDIA Coordinating Center to access the data needed to meet the study objectives. The current study was approved by the Ethics Committee of the Guangdong Provincial People's Hospital.

The present study included participants who had BP measurements on three or more visits between baseline and Year 30, and who underwent comprehensive echocardiography at Year 30. Among 5115 participants, 2271 participants were excluded: 1756 did not attend the year‐30 examination, 502 did not undergo echocardiography at Year 30 and 13 did not have BP measurement data for three or more visits. The final analytic cohort was composed of 2844 participants.

## 3 BP

BP was measured at each CARDIA visit and reported as the average of second and third readings taken by trained personnel using a Hawksley random‐zero sphygmomanometer (0–15 years; Hawksley) or a digital BP monitor (20–30 years; Omron HEM‐907XL; Online Fitness). A calibration study was performed and standardized to the sphygmomanometer measure to remove any potential machine bias. Cumulative systolic BP was calculated for each participant as the sum of the product of mean systolic BP for each pair of consecutive examinations and the time interval between these two consecutive examinations over 30 years of follow‐up. Cumulative diastolic BP was calculated in an analogous manner.

## 4 ECHOCARDIOGRAPHY OUTCOME MEASURES

Comprehensive echocardiography, including two‐dimensional (2D), M‐mode, Doppler, and tissue Doppler, was performed at the year‐30 examination conforming to American Society of Echocardiography guidelines.^16^ Transthoracic echocardiography was performed by well‐trained and certified technicians using a standard machine (Toshiba Medical Systems), with phased‐array transducers from 1.8 to 4.2 MHz, following a standardized protocol across all field centers.

Right ventricular basal diastolic dimension was acquired from a 2D four‐chamber view. Tricuspid annular plane systolic excursion (TAPSE) was measured as the peak excursion of the lateral tricuspid annulus in the apical four‐chamber view using an M‐mode cursor. Tissue doppler imaging was used to assess tricuspid annular peak systolic velocity (right ventricular peak systolic velocity [RVS′]), tricuspid annular early diastolic velocity (right ventricular early diastolic velocity [RVe′]), and tricuspid annular late diastolic velocity (right ventricular late diastolic velocity [RVa′]). The tricuspid regurgitant (TR) jet velocity was measured in triplicate using continuous‐wave Doppler echocardiography. Pulmonary arterial systolic pressure (PASP) was determined from the TR jet velocity using the modified Bernoulli equation, and combining this value with an assumed right atrial pressure of 10 mm Hg (PASP = 4[TR jet velocity]2 + 10 mm Hg). The higher the RVS′ or TAPSE, the better the RV systolic function. The higher the RVe′, the better the RV diastolic function. Left ventricular (LV) diastolic function was assessed by tissue Doppler‐derived mitral annular velocity (*e*′) at the septal and lateral walls. Pulsed Doppler‐derived *E*‐wave from mitral inflow was used to derive the *E*/*e*′ ratio, a surrogate for LV filling pressures. LV end‐diastolic volume and LV ejection fraction were measured by biplane Simpson's method to assess LV systolic function. The latest American Society of Echocardiography guidelines recommended cut‐off values for defining RV systolic and diastolic dysfunctions as follows: RVS’ < 9.5 cm/s or TAPSE < 17 mm/s and RVe′ < 7.8 cm/s.^16^

## 5 COVARIATES

Standardized protocols were used for the collection of height, weight, glucose levels, lipid levels, lung function, and education level at each visit. Demographic characteristics, smoking status, and physical activity levels were self‐reported. Body mass index (BMI) was calculated as weight (kg) divided by height in meters squared. Lung function measurements included FVC (forced vital capacity) and FEV1 (forced expiratory volume in one second).

## 6 STATISTICAL ANALYSIS

Normally distributed continuous variables were presented as mean (SD), while non‐normal data were presented as the median and interquartile range. Categorical variables were presented as counts (percentages). Differences between groups were tested by the *χ* ^2^ test for categorical data, and the independent Student *t* test and Mann–Whitney *U* were used for normally and nonnormally distributed continuous variables, respectively. Multivariable linear regression models were used to assess the association between cumulative BP over 30 years and echocardiographic parameters measured at Year 30 (all as continuous variables). Multivariable models were adjusted for the following traditional cardiovascular disease risk factors. Model 1 is unadjusted; Model 2 included age, race, gender, BMI, current smoking status, educational level, antihypertensive medications, total cholesterol, high‐density lipoprotein, fasting glucose, physical activity, FVC, and FEV1 at Year 30; Model 3 included the variables in Model 2 and left ventricular ejection fraction, *E*/*e*′ septal, *E*/*e*′ lateral, left ventricular mass and left ventricular end‐diastolic volume as LV function. In addition, restricted cubic spline analysis with four knots was used to explore the nonlinear dose–response relationship between cumulative BP over 30 years and echocardiographic parameters measured at Year 30.

For categorical analyses of clinically significant RV dysfunction, binary logistic regression models were performed to estimate the association between cumulative BP over 30 years and clinically relevant RV dysfunction at Year 30. In multivariate logistic regressions, we adjusted for similar covariates, reporting odds ratios (ORs) and 95% confidence intervals (CIs). Potential effect modification by race and sex was assessed.

All analyses were performed using R 3.6.2 software and a *p* < .05 was considered as statistically significant.

## 7 RESULTS

Overall, the study cohort included a total of 2844 participants (mean age: 55.1 ± 3.6 years, 56.8% female, 53.1% White). Table 1 presents the characteristics of the study participants by grouped by sex. Mean values for conventional echocardiographic parameters were within the normal range in mid‐age (43–55 years). Females were more likely to have higher BMI, LDL‐cholesterol, and HDL‐cholesterol and lower systolic blood pressure (SBP), diastolic blood pressure (DBP), cumulative SBP, cumulative DBP, physical activity, FVC, and FEV1. A higher portion of males were current smokers.

**Table 1:** Participant characteristics by gender at year‐30 exam

**Variable**	Total	Male	Female	*p* value
	***N*** = **2844**	***N*** = **1227**	***N*** = **1617**	
Age (years)	55.1 (3.55)	55.1 (3.52)	55.1 (3.57)	.822
White	1509 (53.1%)	687 (56.0%)	822 (50.8%)	.007
BMI (kg/m^2^)	30.1 (6.57)	29.5 (5.26)	30.6 (7.37)	<.001
SBP (mm Hg)	119 (16.4)	121 (18)	117 (22)	<.001
DBP (mm Hg)	73.9 (11.0)	74 (13)	73 (15)	<.001
Cumulative SBP (mm Hg × year)	3313 (391)	3461 (351)	3273 (401)	<.001
Cumulative DBP (mm Hg × year)	2114 (309)	2200 (288)	2093 (304)	<.001
Current smoker	384 (13.7%)	194 (16.0%)	190 (11.9%)	<.001
Educational level (years)	14.9 (1.92)	14.7 (1.96)	14.9 (1.89)	.006
Antihypertensive medications	889 (31.3%)	375 (30.6%)	514 (31.9%)	.511
Total cholesterol (mg/dl)	191 (48)	183 (44)	195 (47)	<.001
LDL‐cholesterol (mg/dl)	108 (44)	108 (44)	110 (42)	.099
HDL‐cholesterol (mg/dl)	61 (249)	50 (19)	64 (24)	<.001
Fasting glucose (mg/dl)	102 (31.2)	106 (35.1)	98.9 (27.4)	<.001
Physical activity (EU)	329 (273)	397 (301)	278 (238)	<.001
FVC (L)	3.33 (1)	4.31 (1)	3.01 (1)	<.001
FEV1 (L)	2.58 (1)	3.27 (1)	2.34 (1)	<.001
RV basal diameter (cm)	3.75 (1)	3.98 (1)	3.54 (1)	<.001
TAPSE (mm)	24.2 (5.50)	23.8 (5.72)	24.0 (5.48)	.183
RVS′ (cm/s)	13.4 (2.62)	13.4 (2.70)	13.4 (2.56)	.701
RVe′ (cm/s)	12.3 (4.77)	11.4 (4.54)	12.3 (4.55)	<.011
RVa′ (cm/s)	14.3 (5)	13.7 (5)	14.6 (5)	<.011
PASP (mm Hg)	30.7 (6.00)	31.2 (5.25)	31.0 (6.00)	.142
LV ejection fraction (%)	59.6 (5.16)	58.3 (5.67)	60.6 (4.48)	<.001
*E*/*e*′ septal	9.22 (2.85)	8.95 (2.71)	9.43 (2.93)	<.001
*E*/*e*′ lateral	7.38 (8.77)	6.75 (2.14)	7.86 (11.4)	<.001
LV end‐diastolic volume (ml)	112.3 (34)	133.0 (33)	104.4 (28)	<.001

*Note*: Data are presented as mean (SD), median (interquartile range), or *n* (%) as appropriate.

Abbreviations: BMI, body mass index; DBP, diastolic blood pressure; *E*/*e*, ratio of early peak diastolic mitral velocity/peak early diastolic mitral annular velocity; FEV1, forced expiratory volume in one second; FVC, forced vital capacity; HDL, high‐density lipoprotein; LDL, low‐density lipoprotein; LV, left ventricular; LVEDV, left ventricular end‐diastolic volume; PASP, pulmonary artery systolic pressure; RV, right ventricular; RVa′, right ventricular peak late diastolic velocity; RVe′, right ventricular peak early diastolic velocity; RVS′, right ventricular peak systolic velocity; SBP, systolic blood pressure; TAPSE, tricuspid annular plane systolic excursion.

Table 2 shows the results for linear regression models examining the association between cumulative BP exposure and echocardiographic RV parameters. In the fully adjusted model, higher cumulative systolic BP was independently associated with lower TAPSE (*β* = −.21, *p* < .05), RVS′ (*β* = −.26, *p* < .01), RVe′ (*β* = −.32, *p* < .01) and higher PASP (*β* = −.71, *p* < .01). Higher cumulative diastolic BP was independently associated with smaller RV basal diameter (*β* = −.03, *p* < .05), lower TAPSE (*β* = −.48, *p* < .01), RVS′ (*β* = −.36, *p* < .01), RVe′ (*β* = −.48, *p* < .01), and higher PASP (*β* = −.32, *p* < .05).

**Table 2:** Relationship of cumulative exposure to BP over 30 years to RV functional parameters

	Cumulative SBP per 1‐SD higher			Cumulative DBP per 1‐SD higher		
	Model 1	Model 2	Model 3	Model 1	Model 2	Model 3
	*β* (95% CI)	*β* (95% CI)	*β* (95% CI)	*β* (95% CI)	*β* (95% CI)	*β* (95% CI)
RV basal diameter (cm)	.08	−.00	−.02	.04	−.03	−.03
	(0.06, 0.10)	(−0.03, 0.02)	(−0.05, 0.01)	(0.02, 0.06)	(−0.06, −0.01)	(−0.05, −0.01)
TAPSE (mm)	−1.87	−.07	−.21	−.43	−.45	−.48
	(−0.34, −0.03)	(−0.27, 0.13)	(−0.45, −0.00)	(−0.58, −0.27)	(−0.65, −0.26)	(−0.69, −0.27)
RVS′ (cm/s)	−.14	−.24	−.26	−.24	−.39	−.36
	(−0.23, −0.04)	(−0.36, −0.11)	(−0.40, −012)	(−0.33, −0.14)	(−0.52, −0.27)	(−0.49, −0.23)
RVe′ (cm/s)	−.61	−.48	−.32	−.66	−.62	−.48
	(−0.73, −0.48)	(−0.63, −0.32)	(−0.50, −0.14)	(−0.78, −0.54)	(−0.77, −0.46)	(−0.65, −0.32)
RVa′ (cm/s)	−.25	−.09	.05	−.27	−.23	−.14
	(−0.39, −0.10)	(−0.28, 0.09)	(−0.16, 0.25)	(−0.41, −0.13)	(−0.45, −0.05)	(−0.33, 0.06)
PASP	1.53	.87	.71	1.29	.39	.32
	(1.23, 1.82)	(0.50, 1.23)	(0.32, 1.10)	(1.00, 1,58)	(0.04, 0.74)	(0.00, 0.72)

*Note: β* represents unstandardized regression coefficients. Model 1: unadjusted; Model 2: Adjusted for age, race, gender, body mass index, current smoking status, educational level, antihypertensive medications, total cholesterol, high‐density lipoprotein, fasting glucose, physical activity, forced vital capacity, and forced expiratory volume in one second measured at Y30 examination. Model 3: Model 2 + left ventricular ejection function, *E*/*e*′ septal, *E*/*e*′ lateral and left ventricular end‐diastolic volume, and left ventricular mass.

Abbreviations: CI, confidence interval; DBP, diastolic blood pressure; *E*/*e*′, ratio of early peak diastolic mitral velocity/peak early diastolic mitral annular velocity; PASP, pulmonary artery systolic pressure; RVa′, right ventricular peak late diastolic velocity; RVe′, right ventricular peak early diastolic velocity; RVS′, right ventricular peak systolic velocity; SBP, systolic blood pressure; TAPSE, tricuspid annular plane systolic excursion.

Results from the binary logistic regression models to examine the association between cumulative BP exposure and RV dysfunction are summarized in Table 3. RV systolic dysfunction (RVS′ < 0.95 cm/s or TAPSE < 17 mm/s) was present in 196 participants, diastolic dysfunction (and RVe′ < 7.8 cm/s) was present in 204 participants. In fully adjusted models, cumulative systolic BP was not related to systolic dysfunction (OR, 1.11; 95% CI, 0.88 − 1.40; *p* = .396). Per 1 SD increase in cumulative systolic BP was associated with a higher risk of diastolic dysfunction (OR, 1.43; 95% CI, 1.14–1.80; *p* < .01), while an increase in cumulative diastolic BP was associated with a higher risk of systolic dysfunction (OR, 1.36; 95% CI, 1.08–1.70; *p* < .01) and diastolic dysfunction (OR, 1.51; 95% CI, 1.21–1.88; *p* < .01).

**Table 3:** RV systolic and diastolic dysfunction at Year 30 predicted from cumulative exposure to SBP and DBP

	Cumulative SBP per 1 SD higher		Cumulative DBP per 1 SD higher	
	OR (95% CI)	*p* value	OR (95% CI)	*p* value
Systolic dysfunction (TAPSE < 17.0 mm or RVS′ < 9.5 cm/s) (*n* = 196)	1.11 (0.88, 1.40)	.396	1.36 (1.08, 1.70)	.007
Diastolic dysfunction (RVe′ < 7.8 cm/s) (*n* = 204)	1.43 (1.14, 1.80)	.002	1.51 (1.21, 1.88)	<.001

*Note*: OR indicates odds ratios. Models adjusted and abbreviations as indicated in Table 2.

**Figure 1:**
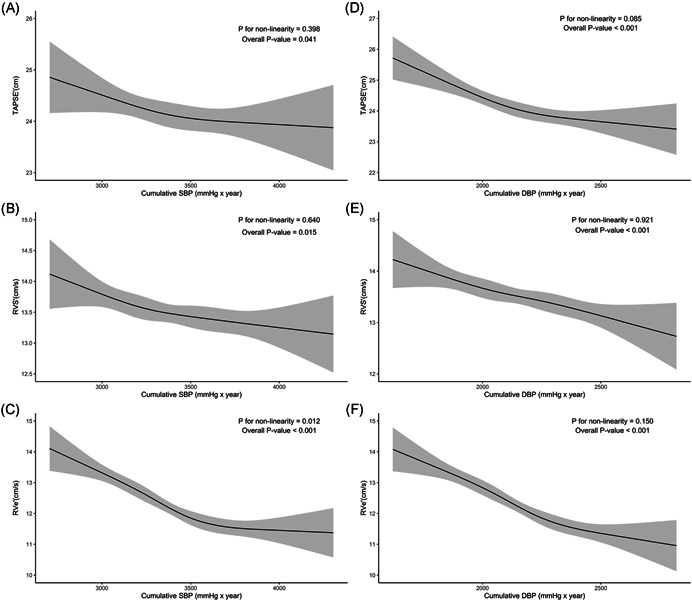
The dose–response relationship between cumulative blood pressure (BP) exposure and right ventricular (RV) echocardiographic parameters. Point estimates (solid line) and 95% confidence intervals (dashed lines) were estimated by restricted cubic splines analysis with four knots. Models adjusted and abbreviations as indicated in Table 2

Figure 1 shows the continuous relationships between cumulative BP exposure and echocardiographic RV parameters based on restricted cubic spline regression models. Linear association was detected between cumulative SBP and TAPSE and RVS′ (*p* for nonlinearity = .398 and .640, respectively). Linear association was detected between cumulative DBP and TAPSE, RVS′, and RVe′ (*p* for nonlinearity > .05). There was no evidence of effect modification by sex and race for the cumulative BP‐RV structure and function associations (all *p* interaction > .05), except for the cumulative BP‐RV basal diameter association which was influenced by sex (*p* interaction = .004 for cumulative SBP and .029 for cumulative DBP).

## 8 DISCUSSION

In this large population‐based study of generally healthy young adults followed over 30 years, several important findings were observed. First, higher cumulative BP exposure from early adulthood was associated with RV dysfunction in middle age. Second, cumulative diastolic BP was more strongly associated with worse RV function parameters compared with cumulative systolic BP. Third, cumulative diastolic BP was associated with smaller RV basal diameter in middle age. Finally, these associations were independent of several confounders, including anthropometric relationships, demographic and clinical variables, as well as the corresponding LV parameters (left ventricular ejection fraction, *E*/*e*′ septal, *E*/*e*′ lateral, left ventricular mass and left ventricular end‐diastolic volume).

A recent paper from CARDIA study reports longitudinal associations of fitness and obesity with RV function, showing that obesity and decline of physical activity were associated with RV dysfunction.^17^ In our study, after adjusting for obesity and physical activity in the multivariable regression models, cumulative BP is still associated with RV dysfunction, suggesting an independent association beyond obesity and physical activity. Studies investigating the associations between cumulative BP and RV structure have shown inconsistent results. Tadic et al.^18^ found that there was no difference in RV basal diameter between controls and untreated hypertensive patients with a mean age of 50 years old. However, Akintude et al.^12^ found that RV end‐diastolic dimensions were lower in hypertensive subjects (age, 57.5 ± 13.33 years) compared with controls (age, 55.9 ± 11.33 years).^12^ In another large study from the Multi‐Ethnic Study of Atherosclerosis, higher BP was associated with smaller RV volumes and lower RV mass in an adult sample without clinical cardiovascular disease.^19^ These results are consistent with our study findings that suggest cumulative DBP is associated with decreased RV basal diameter. Nunez et al.^20^ have previously demonstrated that right ventricular wall hypertrophy occurs in hypertensive subjects. Thereby, it can be deduced that increased thickness of the interventricular septum and RV‐free wall may result in reduced RV dimensions and gradually lead to dilatation in the right heart.

Previous studies on RV function have typically focused on patients with established cardiovascular disease, including heart failure,^21^ myocardial infarction,^22^ or pulmonary arterial hypertension.^23^ The effect of BP on RV function in the general population has not been investigated. In recent years, with the advent of reliable and reproducible echocardiographic measures of RV function and the development of new imaging modalities, the important role of the RV in hypertensive subjects has been increasingly recognized. Pedrinelli et al.^24^ showed that recently diagnosed, untreated young hypertensive patients suffered subclinical RV diastolic dysfunction as assessed by tissue Doppler imaging and RV deformation analysis (performed with 2DE speckle tracking imaging). In another study, Tadic et al.^25^ evaluated the RV functional capacity by 2D conventional echocardiography, speckle tracking, and 3D analysis. They found that RV systolic and diastolic strain rates were reduced in hypertensive participants, whereas 3D EF was decreased in patients with uncontrolled hypertension. In our study, we used M‐mode derived TAPSE and tissue Doppler‐derived RVS′ to assess RV systolic function and tissue Doppler‐derived RVe′ to assess RV diastolic function and found that both cumulative systolic and diastolic BP, even at levels below the hypertension threshold, was associated with RV systolic and diastolic dysfunction in middle age. Furthermore, we observed that both cumulative systolic and diastolic BP were associated with higher PASP on follow‐up. Previous studies reported increased PASP in some patients with early‐stage hypertension, with normal LV filling pressure.^26^, ^27^ This suggests that the activation of common signal pathways may contribute to an increase in both systemic BP and PASP.^27^ To the best of our knowledge, the current study represents the first report on the effect of cumulative BP on RV function. We also found that both systolic and diastolic cumulative BP was more strongly associated with RV function than a single BP reading at Year 30. This suggests the possibility that young adulthood is a critical period in life when exposure to suboptimal BP is particularly important. Additionally, although BP‐lowering strategies may be achieved quickly, subclinical cardiovascular dysfunction(e.g., left ventricular hypertrophy, increased left ventricular mass, and degree of carotid stenosis) has been reported after long‐term exposure to elevated BP in previous studies.^28^

Possible mechanisms responsible for chronically elevated BP and structural RV remodeling may be related to several factors. First, overstimulation of the sympathetic and renin–angiotensin–aldosterone systems, which often present in arterial hypertension, results in the promotion of fibrosis and alterations in the extracellular matrix of the RV.^29^, ^30^ Second, as hypertension‐induced LV hypertrophy commonly affects the interventricular septum, its impact on RV function could potentially translate through left‐to‐right ventricular interactions.^31^, ^32^ Third, LV systolic or diastolic dysfunction leads to elevated LV end‐diastolic pressure, which may cause pulmonary venous hypertension and resultant high PASP, culminating in RV dysfunction.^33^, ^34^

Recently, the role of the right ventricle in a spectrum of CVDs has gained renewed attention.^9^, ^35^ Kawut et al.^9^ found that RV hypertrophy was associated with a risk of clinical HF or cardiovascular death in a multi‐ethnic adult US population without clinical cardiovascular disease at baseline. In a prior CARDIA report, Modin et al.^10^ demonstrated RV systolic function, as assessed by TAPSE, was associated with CVD in the general population. Our results are notable because myocardial modeling and subclinical RV dysfunction are important precursors of incident‐adverse cardiovascular outcomes. Early detection and early, effective, and sustained control of BP may be important to minimize cardiac end‐organ impairments and CVD risk in late life.

## 9 LIMITATIONS OF THE STUDY

This study also has some limitations. First, baseline measures of echocardiographic RV structure and function are not available. Thus, we cannot rule out the possibility of reverse causation. However, the youth and good health of our study population at baseline make it less likely to be related to subclinical dysfunction in baseline cardiac function. Second, echocardiographic data were available in only approximately 50% of the CARDIA participants. Thus there is a potential for selection bias in the observed associations. Third, this study used M‐mode‐derived TAPSE and tissue Doppler‐derived RVS′ as measures of RV systolic function and tissue Doppler‐derived RVe′ as measures of RV diastolic function. Other commonly traditional parameters such as RV Fractional area change, Tei index, speckle tracking echocardiography, and RV *E*/*e*′ (ratio of early peak diastolic tricuspid velocity/peak early diastolic tricuspid annular velocity) were not available for CARDIA patients at Year 30. However, TAPSE, RVS′, and RVe′ are easy to perform, with very low inter‐ and intraobserver variability. Finally, few patients had RV systolic dysfunction. Thus, overadjustment might have been a problem in the multivariable logistic regression.

## 10 CONCLUSION

Cumulative exposure to higher systolic and diastolic BP over 30 years from early adulthood to middle age is associated with incipient RV systolic and diastolic dysfunction in middle age. Exposure to higher diastolic BP levels from young adulthood to midlife is associated with a smaller RV basal diameter in midlife.

## CONFLICTS OF INTERESTS

The authors declare that there are no conflict of interests.

## Data Availability

The data generated and analyzed during the current study are obtained from the CARDIA Coordinating Center (https://www.cardia.dopm.uab.edu/contact-cardia), but restrictions apply to the availability of these data, which were used under license for the current study, and so are not publicly available. The National Heart, Lung and Blood Institute policies governing the data and describing its access can be found online (https://www.cardia.dopm.uab.edu/study-information/nhlbi-data-repository-data).
